# Supplementary material for: The Nutritional Gene Expression Regulation Potential of a Lysolecithin-Based Product
Source: Curr Issues Mol Biol. 2025 Jul 15;47(7):548. doi: 10.3390/cimb47070548 (PMC12293776; doi:10.3390/cimb47070548)
Supplement: Supplementary file 1 [file cimb-47-00548-s001.zip › cimb-3717112-supplementary.pdf]

Supplementary Information

Full list of metabolites that were significantly more and less abundant in response to lysolecithin treatment.

Supplemental Table S1. Comparison of sample and reference genomes

sample	total_reads	total_map	unique_map	multi_map	read1_map	read2_map	positive_map	negative_map	splice_map	unsplice_[illegible]
C_1	90018708	81381602(90.41%)	79509232(88.33%)	1872370(2.08%)	39878106(44.3%)	39631126(44.03%)	39692774(44.09%)	39816458(44.23%)	28842437(32.04%)	50666795(56.[illegible]
C_2	74520142	65786247(88.28%)	64249943(86.22%)	1536304(2.06%)	32267836(43.3%)	31982107(42.92%)	32061226(43.02%)	32188717(43.19%)	23945234(32.13%)	40304709(54.[illegible]
C_3	69712000	62474195(89.62%)	60987448(87.48%)	1486747(2.13%)	30600333(43.9%)	30387115(43.59%)	30453742(43.69%)	30533706(43.8%)	23209004(33.29%)	37778444(54.[illegible]
C_4	77544246	69347904(89.43%)	67632015(87.22%)	1715889(2.21%)	33910987(43.73%)	33721028(43.49%)	33772350(43.55%)	33859665(43.66%)	26020106(33.56%)	41611909(53.[illegible]
C_5	73428410	65328156(88.97%)	63767921(86.84%)	1560235(2.12%)	31985871(43.56%)	31782050(43.28%)	31837081(43.36%)	31930840(43.49%)	24541853(33.42%)	39226068(53.[illegible]
C_6	64014400	57160900(89.29%)	55793722(87.16%)	1367178(2.14%)	27976830(43.7%)	27816892(43.45%)	27846854(43.5%)	27946868(43.66%)	20875151(32.61%)	34918571(54.[illegible]
L_7	69579856	63442041(91.18%)	61954816(89.04%)	1487225(2.14%)	31061177(44.64%)	30893639(44.4%)	30941606(44.47%)	31013210(44.57%)	23479631(33.74%)	38475185(55[illegible]
L_8	56978596	51072040(89.63%)	49882425(87.55%)	1189615(2.09%)	25001251(43.88%)	24881174(43.67%)	24904199(43.71%)	24978226(43.84%)	18511353(32.49%)	31371072(55.[illegible]
L_9	57175152	50828031(88.9%)	49601866(86.75%)	1226165(2.14%)	24896866(43.54%)	24705000(43.21%)	24755556(43.3%)	24846310(43.46%)	18998623(33.23%)	30603243(53.[illegible]
L_10	61564566	55101557(89.5%)	53788095(87.37%)	1313462(2.13%)	26987411(43.84%)	26800684(43.53%)	26849833(43.61%)	26938262(43.76%)	19906569(32.33%)	33881526(55.[illegible]

Supplemental Table S2. Metabolite data that were significantly differentially abundant in negative ionization mode.

HMDB_ID	Compound_name	Chemical Formula	FC(LEX/Control)	Log2(FC)	T-Test
HMDB0059972	4-Hydroxy-5-(3′,4′-dihydroxyphenyl)-valeric acid-O-methyl-O-glucuronide	C18H26O12	0.04	-4.51	0.0025
HMDB0060491	Mycophenolic acid O-acyl-glucuronide	C23H28O12	0.05	-4.36	0.0001
HMDB0260340	Cytidine-5′-diphosphocholine	C14H26N4O11P2	0.05	-4.32	0.0033
HMDB0038866	Quercetagetin 3′-methylether 7-glucoside	C22H22O13	0.07	-3.83	0.0000
HMDB0258916	Tetramethylchromanol glucoside	C20H30O7	0.09	-3.49	0.0000
HMDB0252642	Gardenoside	C17H24O11	0.09	-3.43	0.0000
HMDB0038809	Luteolin 4′-glucoside 7-galacturonide	C27H28O17	0.22	-2.18	0.0002
HMDB0038468	Luteolin 4′-glucoside	C21H20O11	0.28	-1.85	0.0001
HMDB0041144	8-Deoxy-11,13-dihydroxygrosheimin	C15H20O5	0.46	-1.12	0.0001
HMDB0060015	Phenol sulphate	C6H6O4S	0.47	-1.08	0.0000
HMDB0000228	Phenol	C6H6O	0.48	-1.04	0.0002
HMDB0029610	Ascladiol	C7H8O4	2.02	1.01	0.0112
HMDB0035338	Sterebin B	C20H32O5	2.33	1.22	0.0015
HMDB0000784	Azelaic acid	C9H16O4	3.08	1.62	0.0008
HMDB0036199	2-Methoxy-4-(4-methyl-1,3-dioxolan-2-yl)phenol	C11H14O4	3.31	1.73	0.0008
HMDB0031127	5-Hexyltetrahydro-2-furanoctanoic acid	C18H34O3	3.33	1.73	0.0004
HMDB0061914	8-Hydroxyoctanoate	C8H16O3	3.58	1.84	0.0000
HMDB0004667	13-HODE	C18H32O3	3.71	1.89	0.0027
HMDB0010725	3-Hydroxydecanoic acid	C10H20O3	4.05	2.02	0.0000
HMDB0038731	4,7-Megastigmadien-9-ol	C13H22O	4.67	2.22	0.0001
HMDB0030982	6-Ketomyristic acid	C14H26O3	4.77	2.25	0.0003
HMDB0002259	Heptadecanoic acid	C17H34O2	5.44	2.44	0.0003
HMDB0036143	Monomenthyl succinate	C14H24O4	5.66	2.50	0.0001
HMDB0114758	LysoPA(20:2)	C23H43O7P	7.24	2.86	0.0490
HMDB0040668	Blumenol C glucoside	C19H32O7	7.30	2.87	0.0002
HMDB0010387	LysoPC(18:3)	C26H48NO7P	8.08	3.01	0.0242
HMDB0004706	8-Hydroperoxylinoleic acid	C18H32O4	8.68	3.12	0.0002

HMDB0034673	Calamendiol	C15H26O2	9.07	3.18	0.0001
HMDB0006236	Phenylacetaldehyde	C8H8O	10.78	3.43	0.0001
HMDB0062363	6-Hydroxy-3-oxotetradecenoic acid	C14H24O4	11.48	3.52	0.0000
HMDB0000560	Goshuyic acid	C14H24O2	12.26	3.62	0.0000
HMDB0303565	Eucannabinolide	C22H28O8	13.81	3.79	0.0016
HMDB0302703	Eremanthin	C15H18O2	13.83	3.79	0.0001
HMDB0004708	9,12,13-TriHOME	C18H34O5	14.64	3.87	0.0000
HMDB0000672	Hexadecanedioic acid	C16H30O4	15.05	3.91	0.0002
HMDB0004704	9,10-DHOME	C18H34O4	16.17	4.02	0.0002
HMDB0249582	Candoxatrilat	C20H33NO7	16.90	4.08	0.0002
HMDB0000792	Sebacic acid	C10H18O4	19.31	4.27	0.0001
HMDB0014432	Travoprost	C26H35F3O6	20.68	4.37	0.0028
HMDB0038181	Peperinic acid	C10H14O3	25.54	4.67	0.0001
HMDB0002829	Androsterone glucuronide	C25H38O8	39.46	5.30	0.0002
HMDB0254257	Lykurim	C10H24N2O8S2	45.28	5.50	0.0002
HMDB0303749	7-Epi-12-hydroxyjasmonic acid	C12H18O4	49.80	5.64	0.0000
HMDB0260561	MG(PGE1)	C23H40O7	54.36	5.76	0.0004
HMDB0038188	Curcolone	C15H18O3	61.78	5.95	0.0001
HMDB0032960	1-Octen-3-yl primeveroside	C19H34O10	63.19	5.98	0.0002
HMDB0243710	Lisinopril-tryptophan	C27H34N4O5	63.64	5.99	0.0002
HMDB0000394	3-Hydroxytetradecanedioic acid	C14H26O5	76.74	6.26	0.0001
HMDB0039958	4-(4′-O-Acetyl-alpharhamnosyloxy)benzaldehyde	C15H18O7	78.24	6.29	0.0099
HMDB0240698	Daidzein 4′-sulfate	C15H10O7S	78.96	6.30	0.0001
HMDB0030084	Diosbulbinoside D	C25H30O11	88.85	6.47	0.0001
HMDB0038626	Xanthotoxol glucoside	C17H16O9	89.74	6.49	0.0001
HMDB0003306	Phloretin	C15H14O5	90.71	6.50	0.0001
HMDB0036340	Ethyl 7-epi-12-hydroxyjasmonate glucoside	C20H32O9	90.81	6.50	0.0003
HMDB0060120	10-Hydroxy-octadec-enoate-9-glucuronide	C24H42O10	90.94	6.51	0.0027
HMDB0000711	Hydroxyoctanoic acid	C8H16O3	93.79	6.55	0.0001
HMDB0061655	3-hydroxytridecanoic acid	C13H26O3	95.95	6.58	0.0002

HMDB0029349	Neryl rhamnosyl-glucoside	C22H38O10	99.16	6.63	0.0002
HMDB0030680	Diplosporin	C12H16O4	100.06	6.64	0.0001
HMDB0072863	MG(12:0/0:0)	C15H30O4	101.69	6.67	0.0002
HMDB0240372	3-Phenylpropionic acid sulfate	C9H10O5S	106.98	6.74	0.0001
HMDB0003193	Testosterone glucuronide	C25H36O8	107.30	6.75	0.0002
HMDB0037381	Physangulide	C28H42O9	108.13	6.76	0.0002
HMDB0041785	Tyrosol 4-sulfate	C8H10O5S	124.51	6.96	0.0001
HMDB0035209	Cartormin	C27H29NO13	158.01	7.30	0.0000
HMDB0037851	Apigenin 7-sulfate	C15H10O8S	158.41	7.31	0.0002
HMDB0000910	Tridecanoic acid	C13H26O2	165.53	7.37	0.0001
HMDB0006203	5-alpha-Dihydrotestosterone glucuronide	C25H38O8	168.96	7.40	0.0002
HMDB0030737	Capsianoside V	C26H42O10	189.09	7.56	0.0003
HMDB0040390	Erinacine B	C25H36O6	207.94	7.70	0.0005
HMDB0003217	Genistein	C15H10O5	214.38	7.74	0.0002
HMDB0003312	Daidzein	C15H10O4	229.45	7.84	0.0001
HMDB0010343	Ibuprofen glucuronide	C19H26O8	266.67	8.06	0.0001
HMDB0036488	Annuolide C	C15H18O3	293.68	8.20	0.0001
HMDB0240563	Tyrosol glucuronide	C14H18O8	394.72	8.62	0.0001
HMDB0039974	p-Menthane-1,2,8,9-tetrol	C10H20O4	442.75	8.79	0.0001
HMDB0012994	Leukotriene D5	C25H38N2O6S	487.85	8.93	0.0001
HMDB0240527	Homovanillyl alcohol glucuronide	C15H20O9	497.12	8.96	0.0000
HMDB0296943	DG(2:0/5-iso PGF2VI)	C23H38O8	504.56	8.98	0.0001
HMDB0060119	12-O-Glucuronoside-13-hydroxyoctadec-enoate	C24H42O10	735.77	9.52	0.0001
HMDB0260510	MG(PGD1/0:0)	C23H40O7	769.53	9.59	0.0002
HMDB0041413	Marmesin rutinoside	C26H34O13	4897.37	12.26	0.0001
HMDB0240703	Isolariciresinol sulfate	C20H24O9S	10720.49	13.39	0.0001

Supplemental Table S3. Metabolite data that were significantly differentially abundant in positive ionization mode.

HMDB_ID	Compound_name	Chemical Formula	FC(LEX/Control)	Log2(FC)	T-Test
HMDB0035071	Verimol I	$C_{12}H_{14}O_3$	254.98	7.99	0.0000
HMDB0240735	Isolariciresinol glucuronide	$C_{26}H_{32}O_{12}$	207.38	7.70	0.0000
HMDB0011571	MG(18:4/0:0)	$C_{21}H_{34}O_4$	115.04	6.85	0.0003
HMDB0251873	Epothilone A	$C_{26}H_{39}NO_6S$	48.70	5.61	0.0000
HMDB0060121	9-Hydroxy-10-O-glucuronoside-octadecenoate	$C_{24}H_{42}O_{10}$	46.68	5.54	0.0004
HMDB0033153	4′,6-Dihydroxyaurone	$C_{15}H_{10}O_4$	37.80	5.24	0.0002
HMDB0094680	Octaethylene glycol	$C_{16}H_{34}O_9$	6.23	2.64	0.0005
HMDB0244865	1-Benzazepine	$C_{10}H_9N$	3.70	1.89	0.0006
HMDB0006547	Stearidonic acid	$C_{18}H_{28}O_2$	2.95	1.56	0.0019
HMDB0012897	beta-Carboline	$C_{11}H_8N_2$	0.39	-1.37	0.0057
HMDB0258270	Shanzhiside	$C_{16}H_{24}O_{11}$	0.19	-2.42	0.0000
